# Discounting past experience and the utility of memory: an empirical study

**DOI:** 10.1007/s11229-025-04992-x

**Published:** 2025-04-10

**Authors:** Jack Shardlow, Ruth Lee, Patrick A. O’Connor, Christoph Hoerl, Teresa McCormack

**Affiliations:** 1 University of Edinburgh, Edinburgh, Scotland; 2 York St John University, York, England; 3 Queen’s University Belfast, Belfast, Northern Ireland; 4 University of Warwick, Coventry, England; 5 School of Philosophy, Psychology, and Language Sciences, University of Edinburgh, 40 George Square, Edinburgh, EH8 9JX Scotland

**Keywords:** Time biases, Temporal discounting, Utility, Memory, Hedonic preferences

## Abstract

It has been argued that adult humans are absolutely time biased towards the future, at least as far as purely hedonic experiences (pain/pleasure) are concerned. What this means is that they assign zero value to them once they are in the past. Recent empirical studies have cast doubt on this claim, suggesting that while adults hold asymmetrical hedonic preferences – preferring painful experiences to be in the past and pleasurable experiences to lie in the future – these preferences are not absolute and are often abandoned when the quantity of pain or pleasure under consideration is greater in the past than in the future. Research has also examined whether such preferences might be affected by the utility people assign to experiential memories, since the recollection of past events can itself be pleasurable or aversive. We extend this line of research, investigating the utility people assign to experiential memories regardless of tense, and provide – to our knowledge – the first quantitative attempt at directly comparing the relative subjective weightings given to ‘primary’ experiences (i.e., living through the event first-hand) and ‘secondary’ (i.e., recollective or anticipatory) experiences. We find that when painful events are located in the past, the importance of the memory of the pain appears to be enhanced relative to its importance when they are located in the future. We also find extensive individual differences in hedonic preferences, reasons to adopt them, and willingness to trade them off. This research allows for a clearer picture of the utility people assign to the consumption of recollective experiences and of how this contributes to, or perhaps masks, time biases.

## Introduction

It is taken to be relatively uncontroversial that, at least for purely hedonic experiences (see below), people are future biased, resulting in what can be termed *temporally asymmetric hedonic preferences*: they would rather have pain in their past than in their future, and they would rather have pleasure in their future than in their past (see, e.g., Parfit, 1984; Persson, 2005; Prior, 1959). This is true, it is also often assumed, even if the quantity of the purported past pain or pleasure substantially outweighs the quantity of their future equivalents. In the philosophical literature this has sometimes led to suggestions that future biases are absolute (or near-absolute), where this is to say that the value of pains and pleasures is completely (or very heavily) discounted as soon as they are in the past (see, e.g., Heathwood, 2008, esp. pp. 56 − 7; Sullivan, 2018).

Yet, while the notion that people prefer pain to be in the past rather than the future has seemed so self-apparently true that it has been described as a brute fact about human psychology (Heathwood, 2008), the idea that past experiences have no (or virtually no) subjective value, is, at least on the face of it, considerably less intuitively compelling. For example, it is commonplace for people to say that they wish they had not had to undergo a particular unpleasant experience, such as having had a painful dental procedure, even if the current consequences of the event associated with the experience are beneficial. One way of understanding what someone means by such a statement is that, despite the experience being over, they still accord negative utility to it. However, there is also another quite different way to understand such a statement, which is that *the memories* that they still possess of the unpleasant experience have negative utility. Painful memories are themselves unpleasant in part because the painful experience is not simply left in the past; instead, there is a sense in which it continues to plague us – this being Clementine’s motivation for erasing the memories of a painful relationship in the 2004 Charlie Kaufman film *Eternal Sunshine of the Spotless Mind.*

Thus, it is possible that, even if a negative past experience is accorded relatively little (or no) disutility in and of itself because it is in the past, when asked about its subjective value, people also factor in *the memories* left by that experience. Indeed, because memories of the experience can, in principle, go on being experienced in the future, whereas the experience itself is past, any disparity in the (dis)utility accorded to these memories when compared to the original experience, taken on its own, could itself be a manifestation of a future bias. The aim of the current study is to empirically examine whether such disparities do indeed exist, whether they differ for judgements about negative experiences in the past and in the future (Experiment 1) and how strong they may be (Experiment 2).

It should be stressed that our focus throughout is on what, above, we called ‘purely’ hedonic experiences. This qualification is meant to single out pains and pleasures with no further goods or values attached, i.e., devoid of any instrumental value people may sometimes assign to hedonic experiences – e.g., valuing past suffering because it has made one more resilient. (We will discuss one possible worry about the notion of ‘pure’ hedonic experiences in 4.3, below.) In what follows we will typically drop the qualification ‘pure’, though, and speak simply of hedonic experiences.

The paper is structured as follows. In Sect. 2 we briefly review the literature on temporally asymmetrical hedonic preferences and on the utility of memory (2.1) as well as recent empirical work addressing asymmetrical hedonic preferences (2.2). In Sect. 3 we outline two experiments. Experiment 1 (3.1) investigated the relative utility that people assign to primary experiences of pain and to secondary experiences, specifically memories, of that pain, and how these relative utility judgments are influenced by tense. Experiment 2 (3.2) sought to establish whether and at which point people’s relative utility judgments change in the face of an increase in the duration of never to be remembered pain or an increase in the duration of remembered pain. In Sect. 4 we discuss our findings and how they bear on considerations concerning future bias and the utility of memory.

## Background: philosophy, psychology, time, and utility

### Philosophy and the future bias

There is a lively debate about temporally asymmetrical hedonic preferences in philosophy. Theorists are concerned with the significance of such preferences (particularly for metaphysical debates about time: e.g., Pearson, 2018; Prior, 1959), their (ir)rationality (e.g., Dougherty, 2011; Nguyen, 2022; Sullivan, 2018), and their origins (e.g., Maclaurin & Dyke, 2002; Suhler & Callender, 2012). Across these debates, it is often taken as self-evident that people do exhibit temporally asymmetrical hedonic preferences; what is at issue is why people have them and whether they should.

Parfit (1984) provides a thought experiment to illustrate the intuitive appeal of future bias for experiences of pain. You are asked to imagine being in hospital for invasive surgery. You need to be awake during this surgery, for which no anesthetics can be used. As a result, you will undergo excruciating pain during the surgery. Yet, you are given the comfort that patients are administered a post-operative drug that causes them to forget the previous few hours. Hence your situation is as follows: you will undergo and experience an extremely painful operation, but once you receive the post-operative drug you will have no memories of doing so.

It seems intuitively obvious that– all else being equal– people would prefer a shorter painful operation to a longer painful operation. But, according to Parfit, this preference plausibly changes when the two procedures differ in terms of their *tense*. He asks the reader to imagine waking up in hospital not remembering having fallen asleep. Because of the amnesia-inducing post-operative drug, you are unsure of whether you have already had the operation followed by the drug, or whether you are yet to have the operation. You ask a nurse for information. The nurse cannot remember whether you are the patient who had the operation yesterday, in which case it lasted 10 hours, or the patient who will have the operation tomorrow, in which case it will last 1 hour. The nurse goes to find out.

Parfit claims that the difference in tense would override the simple preference for the shorter operation over the longer one in this scenario, and that people would prefer to hear that they had the 10-hour painful operation yesterday, rather than hearing that they will have the 1-hour painful operation tomorrow – i.e., prefer a far greater pain over a lesser pain just because the former is in the past whereas the latter would be in the future. Importantly, they would do so despite the fact that it implies a greater amount of pain in their life as a whole.

As much as the temporal location of the pain is significant in swaying the reader to share Parfit’s intuitions in response to the thought experiment, what we want to focus on here is whether the inclusion of an amnesia-inducing post-operative drug also plays an important role. While the events we remember are in the past, recollecting them is a mental act in which people can engage in the present or future, and which can itself be more or less pleasant or unpleasant– compare the pleasure afforded by remembering a romantic first kiss to the displeasure you endure when remembering suffering an open fracture.

It is likely that Parfit himself introduced the element of the amnesia-inducing drug into his thought experiment in order to isolate specifically the role of tense in preferences. He explicitly acknowledges that memories of pain can themselves be painful and would therefore constitute “an irrelevant and complicating feature” (1984, p. 167) if included in the thought experiment. This raises the question, though, as to how exactly people’s responses might change if the amnesia feature were removed. Would they now prefer to undergo a shorter painful event in the future rather than face the prospect of memories of a greater quantity of past pain? We will discuss research that directly bears on this issue in what follows. We will also adopt the following terminology that distinguishes between primary and secondary experiences: we will take primary experiences to be the actual experiences one has undergone first hand (such as having a painful operation, or an enjoyable romantic kiss), whereas secondary experiences will be taken to be those that involve merely mentally entertaining a primary experience. As outlined by Elster and Loewenstein (1992), secondary experiences can occur either after a primary experience (e.g., remembering a kiss) or in advance of an experience (e.g., anticipating a painful operation). In what follows, we will sometimes simply refer to ‘the experience’ and ‘memory’, where this is shorthand for the relevant primary and secondary experiences respectively.

The idea that secondary experiences have their own (dis)utility, which can affect the value of undergoing any given primary experience, is not a new one. In popular literature, the writing of Proust makes frequent reference to the pleasure (and displeasure) that memories may afford us. In an academic context, Kahneman (1999) stresses the importance of considering the utility of mental acts when evaluating the overall utility provided by any given event, saying that “pleasures and pains [associated with] remembering the past must surely be counted” (1999, p. 6). Both Elster and Loewenstein (1992) and Morewedge (2015) make a similar case and discuss its implications for decision making. Various empirical findings, too, offer reasons to expect memories of pain or pleasure to influence preferences for hedonic goods. For example, people tend to remember emotionally arousing stimuli in a particularly vivid manner (e.g., Rubin & Kozin, 1984; Schaefer & Philippot, 2005). Since events involving pains and pleasures tend to be emotionally arousing, they may often be vividly remembered, and the vividness of a memory may itself affect its influence on a person’s hedonic preferences. Further, a small but growing body of research directly investigates both the extent to which people are future biased and the potential contributing factors to such a bias, including memories of pain; it is to this research that we now turn.

### Existing empirical research on asymmetrical hedonic preferences

Interdisciplinary research has recently begun to empirically examine explicit preferences regarding the temporal location of hedonic experiences, the influence of differences in the quantity of past and future pain or pleasure on such preferences, and (to a limited extent) the question of whether they are modulated by memories of past pain/pleasure.

In one of the first empirical studies in this area, Greene et al. (2021) had participants read a vignette about an astronaut on a long mission during which bland meals are dispensed by the spaceship daily. There is one exception: the spaceship will dispense the astronaut’s favorite meal (positive hedonic value) or most disliked meal (negative hedonic value) on a single occasion during the long journey. During a moment of uncertainty regarding whether the favorite/disliked meal had yet been eaten, participants made a judgment as to whether they would prefer to find out that the meal had been dispensed yesterday, or was due to be dispensed tomorrow. The majority of participants had the expected asymmetrical hedonic preferences, preferring pleasant experiences to be in the future and unpleasant experiences to be in the past. Greene et al. (2022a) used similar vignettes to probe the effects of altering the ratio of past events to future events. Even when the choice was between 10 past events and 1 future event, the majority of participants preferred 10 past negative events to 1 future negative event.

Using a somewhat different vignette, Latham et al. (2021) investigated whether the bias toward the future is cognitively mediated by people’s assumption that they cannot causally influence past events. They constructed a hypothetical scenario in which participants were indeed able to retrospectively change the past and found that this reduced the number of future biased responses. On this basis, Latham et al. suggest that future bias may (largely) result from the fact that people discount events that cannot be influenced, where past events are typically taken to be immutable and future events to be mutable, and thus that the future bias may be one instance of a more general disposition to affectively discount practically irrelevant events. If true, this suggestion might also be thought to have an impact on the connection between future bias and memory: ordinarily, our memories assume that past events are set in stone, and this explains much of their emotional significance; if this is not so, this potentially reduces their emotional significance quite radically.

Lee et al. (2020) used a different task to examine temporally asymmetrical hedonic preferences in both adults and children. (We will here discuss only the adult data.) Participants judged who they would prefer to be – a character who has had a certain pleasant or unpleasant experience in the recent past (e.g., a free meal at a favorite restaurant or a painful injection), or one who will have the same experience in the near future. They also varied the quantity of past pain or pleasure to examine the extent to which any asymmetrical hedonic preferences were absolute. Lee et al. found consistent evidence that, when asked for preferences between otherwise identical hedonic events in the equidistant past and future, adults prefer pleasure to lie in the future and pain in the past, providing a form of baseline measure of future bias. However, perhaps surprisingly, they also found that such temporal preferences were typically abandoned at the earliest opportunity when the quantity of past pain or pleasure was greater than the quantity located in the future.

On the face of it, Lee et al.’s findings are inconsistent with the intuition behind Parfit’s thought experiment, which is that people would prefer pain in the past over pain in the future even if the amount of the former is much larger than that of the latter. There is, however, a key difference between Lee et al.’s (2020) task and Parfit’s thought experiment that might explain this discrepancy: unlike the latter, the cover story in the former made no mention of an amnesia eliminating any memories of past pain/pleasure. As indicated above, under the assumption that at least some hedonic value is attributed to secondary experiences, we may expect that preferences for past painful events over future painful events will be influenced by considerations regarding the pleasure/displeasure of subsequent memories. We might therefore suspect that the role participants take memories to play in their welfare over time may have masked the strength of an underlying future bias, whether or not it is absolute.

For this reason, Lee et al. (2022) carried out a further study intended to probe the relative weight that children and adults give to the temporal location of painful events and to whether those events will be remembered. (Again, we will here only discuss the adult data.) In a series of experiments, participants were first presented with a vignette requiring them to judge which state of affairs they would hope to discover while in a temporarily disoriented state: that they will experience 1 painful event in the future, or that they have already experienced 10 painful events in the past. In the first experiment, the vignette employed a cover story involving amnesia for the period of time encompassing the painful events, which ruled out a role for memory of pain in informing preferences for the temporal location of painful events. In two subsequent experiments, the authors examined the weight accorded to memories of pain by directly comparing conditions in which the protagonist was said to either have permanent or only temporary amnesia. When adults considered the prospect of past or future experiences devoid of any trace in memory (Amnesia condition), the majority hoped for 10 painful events in their past over only 1 such event in their future, consistent with Parfit’s (1984) intuitions about the strength of asymmetrical hedonic preferences. However, when adults’ memories of the last week were said to be returning shortly (No Amnesia condition), they showed no clear preference between 10 painful events in their past over only 1 such event in their future. Lee et al.’s findings strongly suggest that considerations about the memories caused by painful events contributed to the propensity of some adults in the No Amnesia condition to report a preference for undergoing a lesser amount of first-hand pain in the future over having already undergone a larger amount of first-hand pain in the past.

Lee et al.’s (2022) findings in the Amnesia condition are at least compatible with the idea that many people have the types of asymmetrical hedonic preferences sometimes ascribed to them in the philosophical literature – i.e., that they are absolutely (or near-absolutely) future biased. Interestingly, the comparison between the Amnesia and No Amnesia condition findings also suggests that people do not simply focus on primary experiences when expressing asymmetrical hedonic preferences; they also attribute significant weight to secondary experiences, such as the memories they want to live with (or without, as the case may be). That is, for at least some people, memories appear to be a key modulating factor in their preferences regarding the temporal locations of hedonic goods. For some people, the disutility attached to particular secondary experiences – such as future episodes of recollecting painful past events – can outweigh the disutility of living through shorter future painful events first-hand; thus, considerations concerning the consumption of secondary experiences appear to play an important role in people’s past-future hedonic preferences.

### The relative utility of primary versus secondary experiences

While Lee et al.’s (2022) findings suggest that the availability of the memory of pain may influence people’s temporally asymmetrical hedonic preferences, their study does not directly address the question that we began with, which concerns the relative utility of a past experience versus memory for that experience. None of the decisions people made in Lee et al.’s study could have been straightforwardly based simply on a direct comparison of these two potential sources of (dis)utility, because people were always choosing between options that differed in tense (primary experiences located either in the past or the future). Directly assessing the relative utility of past experiences as compared with memories of such experiences would require the choice to be between options involving the same tense that vary only in the availability of memory, such as 1 hour of a painful past experience that can subsequently be remembered versus 10 hours of a painful past experience that cannot be remembered. It is these sorts of choices that we examined in Experiment 1 and Experiment 2.

We have suggested that a difference in relative utility of past experiences versus memories for such experiences can itself be understood as a manifestation of a specific kind of future bias, because whereas the primary experience is located in the past, memories of those experiences will in principle remain available in the future. However, examining this relative utility may also be of interest independently of the issue of future bias per se, particularly in the context of decision making (Elster & Loewenstein, 1992; Morewedge, 2015). It has been theorized that in deciding whether to undergo a particular experience (e.g., which holiday to take), people may consider what memories that experience might provide, and moreover the impact of the experience on existing memories (Morewedge, 2015). For example, there is some evidence that people will forgo actual pleasant experiences in order to preserve existing pleasant memories (Zauberman, Ratner, and Kim, 2009). People are also prepared to forgo a monetary reward in order to engage in remembering positive versus neutral events (Speer, Bhanji, and Delgado, 2014). These findings strongly suggest that people do accord utility to memories, but they do not directly address the question of the relative utility of a past experience versus memory of that experience. Moreover, for considerations of such relative utility to feature in many types of future-oriented decision making, people would need to anticipate such utility differences in advance of making their decision. If people are future biased, the utility attached to experiences in the future will be different to that attached to past experiences, and this is likely to impact the relative utility of primary versus secondary (specifically memory) experiences. Moreover, mentally anticipating a primary experience is itself a secondary experience (Elster & Loewenstein, 1992), and it is possible that in advance of an aversive experience, people will focus more on the looming dread stemming from the secondary experience– i.e., of anticipation (Berns et al., 2006)– rather than on the subsequent availability or otherwise of memory experiences. Thus, there are reasons to suspect that there may be different preferences when people are considering experiences versus memories of such experiences in the past when compared with the future; Experiment 1 addressed this specific issue.

## Current study

The experiments reported in what follows were designed to expand on the studies by Lee et al. (2020) and Lee et al. (2022). To briefly recap the most relevant results from these studies: Lee et al. (2020) found that (all else being equal) adults prefer pain to lie in the past, though such temporal preferences were typically abandoned at the earliest opportunity when the quantity of past pain was greater than the quantity located in the future. Lee et al. (2022) investigated further how responses of this type were modulated by considerations as to whether participants would or would not remember the painful events in question, and found a preference for 10 painful events in the past over just 1 such event in the future only when participants were told that no memories of the past events would ever return. As mentioned, the set-up of neither study allowed for a direct investigation of the relative disutility of painful events and memories of those events. The aim of the current study was to carry out such an investigation.

In Experiment 1, participants were presented with scenarios in which they were said to undergo a painful event that they could subsequently either remember or not; in each case, both scenarios were located either in the past or in the future, allowing us to investigate to what extent participants’ preference judgements between the scenarios interacted with the temporal locations of the relevant events. We further probed whether the way that a preference judgement is obtained – by framing it as a hypothetical choice, or by asking for preferences about a pending piece of news over which participants have no agency – influences responses. As we return to in the discussion of the findings below, we did this is the light of current discussion in experimental philosophy over whether the method of obtaining preference judgments– as choices or as preferred ‘news’ – potentially impacts patterns of performance (Greene et al., 2024). In Experiment 2 we sought to replicate our key findings while giving participants an opportunity to express indifference, and asked how quickly participants’ relative (dis)utility judgments change as a function of an increase in the duration of remembered or never to be remembered pain.

### Experiment 1

Experiment 1 examined the relative utility that people assign to memories of pain and the magnitude of pain experienced first-hand, by asking participants to express a preference between 10 hours of pain that will never be remembered and 1 hour of pain that will be remembered. Some participants were told that the painful event is in the past, and others that it is in the future. Of primary interest was whether the past location of the experience of pain would render its duration a less important influence on participant preferences than subsequent memories of it. We also wished to know whether participants’ preferences were influenced by the framing of the scenarios that they read: a hypothetical choice, or a passive preference pending a piece of news that they will shortly receive. Finally, we were interested to examine the interaction between the temporal location of the scenario (past or future) and its framing (choice or news).

Power analyses using G*Power (Faul et al., 2007) were conducted for binomial tests with alpha and power at the conventional levels of .05 and .8, respectively, presuming strong temporal preferences. They yielded a minimum required sample size of *N* = 30.

#### Method

**Participants.** 325 adults (*M* = 34.68 years, *SD* = 12.89, 177 males) participated. Participants were allocated randomly between the Future Choice (79), Future News (84), Past Choice (81), and Past news (81) conditions. All participants indicated that English was their native language. Data from an additional 76 adults were collected, but not used as a result of failing check questions during the study (further details are given below). Adults were recruited via the Prolific subject pool (Peer et al., 2017) and received compensation of £1 (UK pounds). Ethical approval for this and all further reported experiments was received from the research ethics committee of Queen’s University Belfast, protocol number EPS21_124, titled ‘The utility of memories in the past and future’.

**Design, materials, and procedure.** Adults completed the experiment online using their own computers or mobile devices. The experiment was presented using Qualtrics software (Qualtrics, Provo, UT). The experiment comprised of an asymmetrical Preference task, in which participants were shown a vignette and were asked to indicate their preference regarding the treatment they receive for a hypothetical disease. Participants were randomly assigned to one of four groups (Past Choice, Past News, Future Choice, or Future News).

**Fig. 1:**
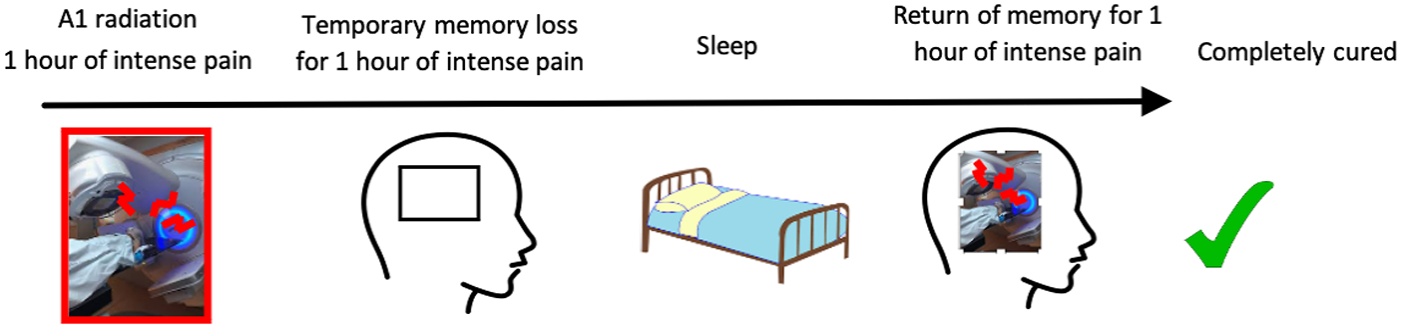
Illustration provided to participants of the course of A1 radiation treatment

**Fig. 2:**
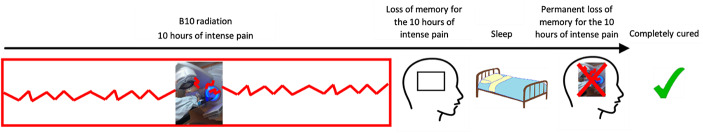
Illustration provided to participants of the course of B10 radiation treatment

**Preference task.** Participants read a short text (see materials available at https://osf.io/vy8j9/) about a hypothetical scenario in which it is discovered that while they currently have no symptoms, they have a brain disease called Denbora Syndrome, which requires painful radiation treatment to avoid future serious health consequences. Participants were told that Denbora Syndrome can take two forms, and that it is not yet known to them which form they are suffering from. Neither type of Denbora Syndrome has more severe health consequences, nor higher risk of a bad outcome, than the other, but they require different types of radiation therapy, both of which are 100% effective. Denbora Syndrome Type A requires A1 radiation: 1 hour of intensely painful radiation therapy, causing temporary loss of memory for that hour once it is over (though no loss of memory for any other time). However, memory for the hour of pain returns after a night’s sleep. Denbora Syndrome Type B requires B10 radiation: 10 hours of intensely painful radiation therapy, causing permanent loss of memory for those 10 hours once they are over (but no loss of memory for any other time). The memory of the 10 hours of pain never returns. These two courses of events were summarized for participants in timeline graphics (see Figs. 1 and 2).

Participants then answered eight questions serving as checks on their understanding. Four of these check questions were accompanied by the illustrations of the course of A1 and B10 radiation shown in Figs. 1 and 2, such that participants could answer the questions based on re-reading the information in the graphic. These questions concerned whether any memory of the pain would exist after waking up the next day, and how long the pain lasts. Participants who failed one or more of these checks on the first attempt were removed from the study. For the other four check questions (regarding the long-term health consequences of both types of Denbora Syndrome, whether either radiation therapy carried a higher risk of long-term damage to one’s health, which treatment is more effective, and whether patients feel pain during the therapy), key information was reiterated to participants who failed the check, and for those participants the check was subsequently repeated. The data of participants who failed one or more of these checks on this second attempt were removed from the study.

In the Past conditions (Past Choice; Past News), the participant was then asked to imagine themselves as a protagonist who had a test last night to discover which type of sickness they have (Denbora Syndrome Type A or Type B), which in turn dictated their treatment. The participant was then said to be returning to the ward after the treatment. Regardless of the type of Denbora Syndrome the participant had and the type of radiation therapy with which it was treated, they were said currently to have amnesia for the period of the treatment. This means they do not remember the test result (which type of sickness they have), nor whether, as a result of their diagnosis, they had the 1- or the 10-hour treatment. They do not yet know whether their memory will return.

In the Future conditions, the protagonist (participant) has not yet been treated; in fact, they have not yet been informed of the test result that will reveal which form of the sickness they have. Therefore, they do not know whether they will be treated with the 1-hour painful radiation therapy, which will be recalled shortly after it takes place, or the 10-hour, never to be remembered, painful therapy.

At the conclusion of the vignette, participants in the Future conditions were informed that they stayed in hospital overnight (in advance of treatment) and have just woken up, and participants in the Past conditions were informed that they have not yet had the chance to sleep. In all four conditions, participants have not yet been informed whether they have Denbora Syndrome Type A or Type B, and hence whether they have been scheduled for A1 or B10 radiation (Future condition), or have been treated with A1 or B10 radiation (Past condition). Participants in the Past Choice and Future Choice conditions were asked to make a hypothetical choice about ‘who they would rather be’: the patient on the ward who had/will have the 1-hour treatment that will be recalled, or the patient who had/will have the 10-hour treatment that will never be recalled. Participants in the Past News and Future News conditions were asked what they ‘hope to hear’ when the nurse returns: that they had/will have the 1-hour treatment that will be recalled, or that they had/will have the 10-hour treatment that will never be recalled. Finally, participants were directed to a free-text question asking the reason for their preference (in the Past News and Future News conditions, ‘Why do you hope to hear this?’ and in the Past Choice and Future Choice conditions, ‘Why would you rather be the patient who…’).

**Data scoring and analysis.** Participants’ binary categorial choices on the Preference task were recorded. Dropped trials arising from failure on the check questions yielded slightly different *N*s across conditions for the analyses below.

#### Results from Experiment 1

**Fig. 3:**
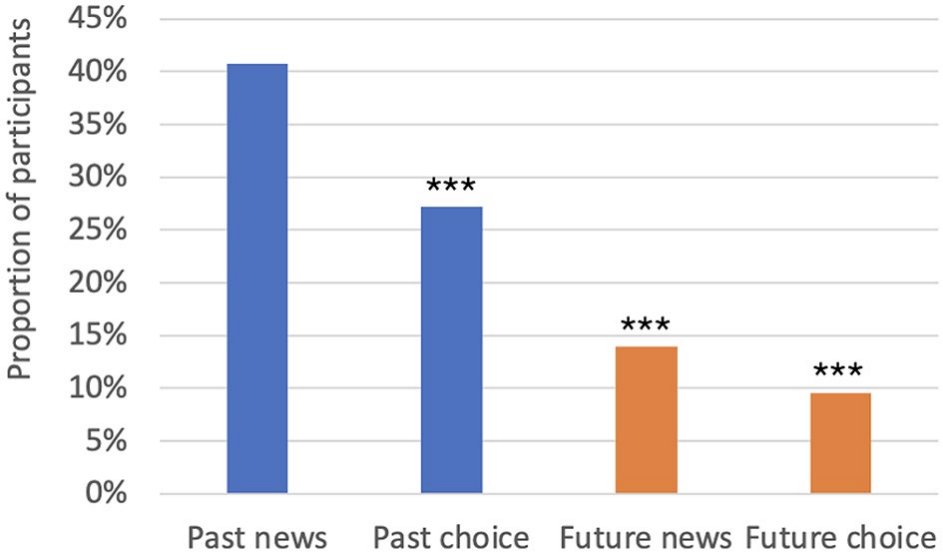
Proportion of participants choosing 10 hours of past pain that will never be remembered, Experiment 1. *Note.* * *p* < .05; ** *p* < .01; *** *p* < .001

Results from the Preference task are reported in Table 1; Fig. 3, where they are shown as a proportion of participants who preferred 10 hours of pain that will never be remembered.

**Table 1:** Results of 2-tailed binomial tests against chance, Experiment 1. Frequencies represent the number of participants who chose 10 hours of pain that will never be remembered

Condition	*n*	Frequency	95% CI	*p*
Future News	79	11	(.07,.24)	<.001*
Future Choice	84	8	(.04,.18)	<.001*
Past News	81	33	(.30,.52)	.119
Past Choice	81	22	(.18,.38)	< .001*

*Note.* Participant numbers vary across trials because data from participants who failed comprehension check criteria were removed on the relevant trial

* Significantly different to chance in the direction of preference for 1 hour of pain that will later be remembered

We first examined participants’ preferences against chance levels using two-tailed binomial tests, separately for each condition (Future News, Future Choice, Past News, Past Choice). Regardless of whether the question about their preference was framed as a choice (who they would rather be) or piece of news (what they hope to hear), participants who read about a scenario located in the future (Future Choice, Future News) preferred 1 painful event that will be remembered to 10 painful events that will never be remembered at a rate above chance. Among participants who read a scenario located in the past, those who encountered the choice framing (Past Choice) preferred 1 painful event that will be remembered at a rate above chance, whereas the preferences of those who encountered the news framing (Past News) were at chance.

To investigate the effect of Tense (Past or Future) and Framing (News or Choice) in comparative terms, we submitted the data to a logistic regression. While a greater proportion of participants preferred 10 painful events that will never be remembered when the scenario was framed as News than when the scenario was framed as Choice, this effect did not reach significance (*p =*.069). There was a main effect of Tense (Wald *χ*^*2*^ (1) = 14.79, *p* < .001, *b* = 1.51, *SE* = 0.40), demonstrating that participants were more likely to prefer 10 painful events that will never be remembered in the Past than in the Future condition (Exp(*B*) = 0.22). There was no interaction between Tense and Framing (*p* = .591).

Participants’ free-text responses to our question about the reason for their preference, ‘Why do you hope to hear this?’ (Past News, Future News conditions) and ‘Why would you rather be the patient who…’ (Past Choice, Future Choice conditions) were not formally analyzed, but were scrutinized for broad categories of response; these informed forced-choice questions that were given to participants in Experiment 2, described below.

#### Discussion of Experiment 1

Experiment 1 suggests that tense has a bearing on preferences: the temporal location of a painful event appeared to influence the relative disutility assigned to its magnitude and subsequent memories. When participants considered news of hypothetical past pain, responses were at chance between 10 hours of never to be remembered and 1 hour of to be remembered pain, suggesting individual differences in the relative weighting assigned to the disutility of memory of pain and the disutility of the (magnitude of) pain experienced first-hand. That is, the fact that at the group level performance did not differ from chance suggested that the sample was roughly evenly split between participants who accord more disutility to memory for pain than to the past experience of pain itself and participants who accord more disutility to the primary experience. However, when considering news of hypothetical future pain, a clear preference for 1 hour of pain to be remembered over 10 hours of pain never to be remembered was evident, suggesting a stronger weighting for the disutility of the magnitude of primary pain relative to the disutility of a future memory of pain.

Why might the disutility of the memory of pain be enhanced, relative to the magnitude of the experience of pain itself, when the painful event is located in the past? We have suggested that, in the case of past experiences, this type of relative disutility of the secondary experience (remembering the pain) compared to the primary experience can be interpreted as a manifestation of a type of future bias, because, whereas the specific experience of pain is fixed in the past, memories can at least in principle re-occur in the future. However, when people were making choices for the future rather than the past in Experiment 1, both the experience of pain and the subsequent memories of such pain (or lack thereof) were located in the future. The relative lack of disutility accorded to future memories of pain in this condition means that participants were instead largely according disutility to either the future primary experience of pain itself, and/or to the unpleasant secondary experience of anticipating the looming pain during the time period before the treatment occurred. That disutility would be assigned to the secondary experience of anticipation is consistent with psychological research indicating that people are highly motivated to avoid dread that occurs when waiting for pain, sometimes to the extent of choosing to undergo a more painful experience immediately rather than have to wait in anticipation of a somewhat less painful experience (Berns et al., 2006), even when outcomes are hypothetical, as in the current study (Löckenhoff et al., 2016). As things stand, our findings do not allow us to infer whether disutility was being assigned to the future experience per se or to the anticipation of it (or both); we return to this issue in the General Discussion.

The framing of response options as a choice or as a piece of anticipated news did not have any direct bearing on this effect of tense, but it did appear to influence responses in absolute terms. When waiting for news, comparison of participant responses against chance differed across Past (at chance) and Future (significant preference for 1 remembered event) conditions, whereas when making a hypothetical choice, participants preferred 1 remembered event regardless of whether the scenario played out in the past or the future. One explanation of this difference may be that a sense of (albeit hypothetical) agency (Latham et al., 2021) over past events leads participants to question the status of memories of those events, or to represent the events themselves as in some sense not set in stone, thereby making potential memories of those events less of a pertinent consideration. Introducing a sense of agency over past events may also lead people to adopt an impersonal stance, which focuses on the influence of one’s choice on one’s life as a whole, rather than a future-facing stance focusing solely on the remainder of one’s life. Adopting such an impersonal stance might lead participants to focus on the displeasure of a greater magnitude of pain in the primary experience, regardless of its temporal location.

We have suggested that the findings of the Past News condition (chance-level performance) can be interpreted as evidence that participants in that condition are roughly evenly split between those who assign more disutility to memories of past pain than to the past painful experience itself, and those for whom the reverse is true. However, one important issue with the design of Experiment 1 is that there is no way to tell whether some participants were in fact indifferent between the options presented to them and, as a result, selected a ‘preferred’ state of affairs at random. For instance, some participants may have had roughly equally weighted preferences between the alternatives presented to them. That is, they may have been indifferent because the particular 1:10 ratio of remembered to never to be remembered pain used in Experiment 1 happened to strike them as evenly weighted. Alternatively, they may have had difficulty weighing the relative utility of primary and secondary experiences because of some perceived qualitative difference between them; or they may have had a sense that the two alternatives are incommensurable– i.e., that there simply is no yardstick by which we can fruitfully compare the (dis)utility of primary and secondary experiences. In Experiment 2 we addressed these possibilities directly, while narrowing our focus to past events and framing our question about preferences in terms of a wait for news. We also examined the question of the point at which a change in the relative duration of never to be remembered and remembered pain might induce participants to alter their initial preference.

### Experiment 2

In Experiment 2 we replicated part of Experiment 1 (the Past News condition) but we made some alterations to the wording used in the task in order to ensure it was as simple as possible and that participants understood the choice they were faced with. In addition, we made some modifications to allow us to better interpret the findings. First, we provided an option that allowed participants to explicitly indicate indifference between 10 hours of pain never to be recalled and 1 hour of pain soon to be recalled. Second, we sought to establish whether and at which point an increase in the duration of never to be remembered pain (where participants’ initial preference was for 10 hours of never to be remembered pain, or they were indifferent) or an increase in the duration of remembered pain (where the initial preference was for 1 hour of remembered pain, or they were indifferent) would result in a change of preference. This allowed us to get a better sense of the magnitude of any relative difference in (dis)utility between memory and the painful past experience itself. Third, we asked participants to make forced-choice indications of the most important reason informing their preference, in the hope that it might yield some useful (if tentative) insights into the basis for participants’ judgments. For example, it could be that participants do accord substantial disutility to painful memories but they also have a strong preference for having intact memories of the past (good or bad) that governs their decisions and masks such disutility. Finally, we examined the role of the intensity of participants’ memories of their worst ever real-life pain. Living with memories of extreme pain might perhaps increase the disutility accorded to memories of pain in general. Conversely, if the phenomenology of experiential memories of extreme pain tends to be substantially qualitatively different from the phenomenology of pain undergone first-hand, then participants with more experience of undergoing extreme pain, and thus of this contrast, may assign less disutility to memories of pain relative to undergoing painful events first-hand.

#### Method

**Participants.** 89 adults (*M* = 41.18 years, *SD* = 16.09, 31 males) participated. All participants indicated that English was their native language. Data from an additional 11 adults were collected, but not used as a result of failing check questions during the study. Adults were recruited via the Prolific subject pool (Peer et al., 2017) and received compensation of £1.25 (UK pounds).

**Design, materials, and procedure.** Materials were drawn from those used in the Past News condition of Experiment 1; we chose not to use the ‘Past Choice’ condition to avoid the possibility that participants take themselves to have hypothetical agency over the past, which may lead them to question the status of any supposed memories. The experiment differed from Experiment 1 in the following ways: the simplification of language, the addition of a ‘No preference’ option to the Preference task, and the replacement of the free-text question regarding the reason for participants’ preference by one or more forced-choice Reason questions. We also added two tasks. A Trade-Off task posited an increase in the duration of the pain delivered by the treatment that participants had originally chosen during the Preference task (A1 or B10 radiation therapy). Participants were asked to consider this change to the scenario that they had initially preferred, and to indicate a point within a specified range of pain durations at which they would reconsider their original preference. In order to explore whether the intensity of past experiences of real pain is associated with the assignation of disutility to painful memories, we also asked participants to indicate the intensity of the worst pain that they had ever experienced.

**Preference task.** The Preference task and accompanying check questions were identical to those used in Experiment 1, save for minor changes to phrasing intended to support participants’ memory for the salient details of the scenarios (see materials available at https://osf.io/vy8j9), and the addition of a ‘no preference’ option at test.

**Reason questions.** The forced-choice Reason questions were presented immediately after participants indicated their response to the Preference task. We asked participants’ reason for their answer (e.g., “Why do you hope to hear that you have Denbora Syndrome Type A, and were treated with A1 radiation? Choose the statement that is closest to your main reason”), and presented several candidate explanations, which differed as a factor of the preference that participants indicated during the Preference task (henceforth, ‘first-tier’ Reason questions). Certain responses prompted an additional (henceforth, ‘second-tier’) forced-choice Reason question to further clarify participants’ main reason for their preference. Reason questions are summarized in Tables 3, 4, 5 and 6.

**Trade-Off task.** Following the forced-choice questions participants were reminded of the two possible outcomes of the scenario described in the Preference task, and of their own stated preference. Participants were then asked to consider a different scenario, identical to the initial vignette save for the duration of the treatment that they originally preferred. If a participant had responded during the Preference task that they preferred 1 hour of remembered pain, they were told that in this different situation, Denbora Syndrome Type A is treated not with A1 radiation, but with A-x radiation. A-x radiation was said to work just like A1 radiation, save that it lasts not for 1 hour, but for between 2 and 10 hours. If a participant had responded during the Preference task that they preferred 10 hours of never to be remembered pain, they were told that in this different situation, Denbora Syndrome Type B is treated not with B10 radiation, but with B-x radiation. B-x radiation was said to work just like B10 radiation, other than lasting not for 10 hours, but for between 20 and 100 hours. The alternatives represented by the new situation were then summarized to participants.

Participants who had initially preferred 1 hour of remembered pain were asked “How many hours would A-x radiation treatment have needed to last for you to hope that you had Denbora Syndrome Type B, rather than Type A, and so had been treated with B10 radiation therapy?” They were presented with a sliding scale representing hours of pain, starting at 2 hours and ending at 10 hours, which allowed them to select any duration between these limits. Participants who had initially preferred 10 hours of never to be remembered pain were asked “How many hours would B-x radiation treatment have needed to last for you to hope that you had Denbora Syndrome Type A, rather than Type B, and so had been treated with A1 radiation therapy?” They were presented with a sliding scale representing hours of pain, starting at 20 hours and ending at 100 hours, which allowed them to select any duration between these extremes. Regardless of the treatment that participants had preferred during the Preference task, they were also offered the option of ticking a ‘Never’ box if they judged that, in the new scenario described in the Trade-Off task, they would never switch from their initial therapeutic preference.

Participants who had selected the ‘No Preference’ option in the Preference task answered two further questions. They were first reminded of their response, and then of the two possible outcomes of the scenario described in the Preference task: diagnosis with Denbora Syndrome Type A or B, leading to treatment with A1 or B10 radiation, respectively. Finally, they read and responded both to the Trade-off question seen by participants who initially preferred 1 hour of remembered pain, and to the Trade-off question seen by those who initially preferred 10 hours of never to be remembered pain (see above). They were also offered the option of ticking a ‘No Preference’ box if they had no preference.

**Pain memory question.** Finally, we asked participants to recall the worst pain that they had ever experienced, no matter whether it lasted a short or a long time, to take a moment to remember it, and then to choose a number that best described the pain at its worst. Following the Brief Pain Inventory (short form: Cleeland & Ryan, 1991), participants answered on a scale of 1 (no pain) to 10 (pain as bad as you can imagine).

#### Results from Experiment 2

**Fig. 4:**
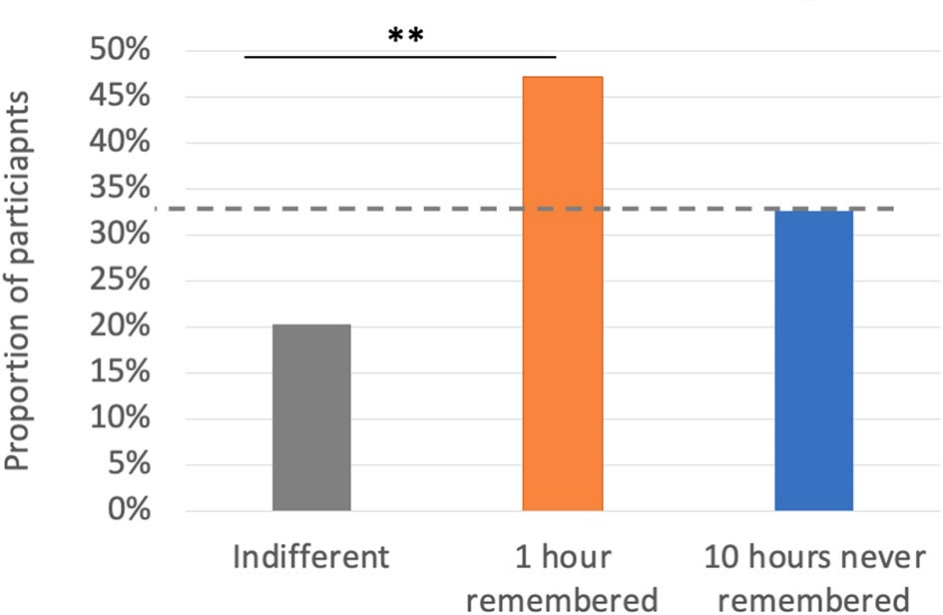
Results of Preference task against chance, set at 33%, Experiment 2. *Note.* * *p* < .05; ** *p* < .01; *** *p* < .001

Forty-two participants (47%) preferred 1 hour of remembered pain; 29 participants (33%) preferred 10 hours of unremembered pain; and 18 participants (20%) were indifferent. We examined these preferences against chance (set at .33) using a chi-square test of goodness of fit. The distribution of participants preferring each option differed from chance, *χ2*(2) = 9.73, *p* = .008 (Fig. 4). Bonferroni-corrected two-tailed binomial tests using an adjusted alpha level of .016 per test (.05/3) demonstrated that a significantly larger proportion of participants preferred 1 hour of remembered pain than were indifferent (*p* = .003). This was not the case when comparing the proportion of indifferent participants with those who preferred 10 hours of never to be remembered pain (*p* = .144), nor when comparing the proportion of participants who preferred 1 hour of remembered pain with the proportion who preferred 10 hours of never to be remembered pain (*p* = .154).

**Fig. 5:**
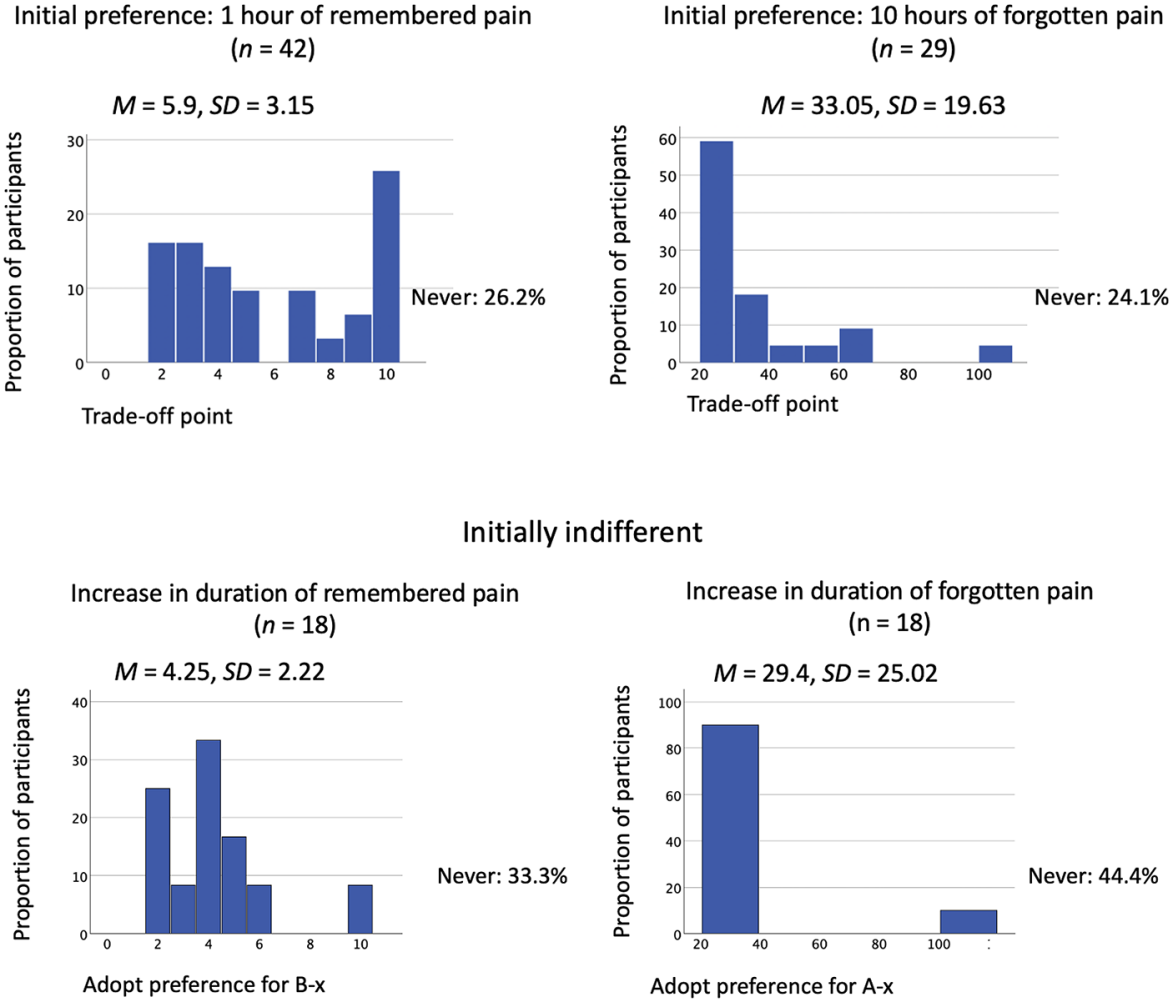
Proportion of participants prepared to switch in the Trade-off task, Experiment 2. *Note*. *n* includes participants whose response was ‘Never’

The pattern of participant responses to the Trade-off task is shown in Fig. 5, stratified by initial preference. Of those participants who expressed an initial treatment preference, regardless of whether it was for 1 hour of remembered pain or 10 hours of never to be remembered pain, approximately one-quarter indicated that they would never switch their preference to the other treatment. The proportion of participants with an initial preference for 1 hour of remembered pain who switched their preference immediately (at 2 hours of remembered pain) was approximately 12%, whereas for those with an initial preference for 10 hours of never to be remembered pain, it was approximately 60% (including those who switched at a point up to and including 25 hour of never to be remembered pain). Approximately one-quarter of participants who initially preferred 1 hour of remembered pain indicated that they would switch their preference only at the maximum possible new duration of their initially preferred treatment (10 hours, thus equaling the duration of B10 radiation); for participants who initially preferred 10 hours of never to be remembered pain, approximately 5% indicated that they would not switch unless the new duration of this treatment was said to be at its maximum (100 hours).

Of those participants who were initially indifferent, 16.7% adopted a preference at the minimum possible new duration of A-x radiation (2 hours), and 38.9% did so at the minimum possible new duration of B-x radiation (20 hours). A large minority indicated that they would never adopt a preference (33.3% in the case of A-x radiation, and 44.4% in the case of B-x radiation).

To investigate the effect on participants’ treatment preferences of the intensity of the worst real-life pain that participants had ever experienced, we submitted the data to an exploratory multinomial logistic regression, which demonstrated that pain intensity did not predict treatment preference (*p* = .273). There was no effect of age on preference (*p* = .389), nor on whether or not participants chose to ever trade off their initial preference (*p* = .400). Participants’ responses to the real-life Pain question are described in Table 2, stratified by response to the Preference question. Responses to Reason questions, first-tier and second-tier, are summarized in Tables 3, 4, 5 and 6. Because of the small numbers of participants choosing some options, we only report second-tier responses when more than 10 participants selected an option that led to a second-tier question.

**Table 2:** Responses to real-life Pain question by response to Preference task, Experiment 2

Preference	*n*	*M (SD)*	Min	Max
1 hour of remembered pain	42	7.50 (1.99)	0	10
10 hours of never to be remembered pain	29	6.72 (1.98)	2	9
Indifferent	18	7.00 (2.35)	1	10

**Table 3:** Responses to first-tier Reason question for a preference for 1 hour of remembered pain, Experiment 2 (*n* = 42)

Reason	Frequency	%
It is less unpleasant to have had fewer total hours of pain	9	21.4
A smaller number of hours of pain that I will remember would cause less unpleasantness for me than a larger number that I won’t ever remember	8	19.0
10 hours of pain would be hard to bear	15	35.7
Remembering the pain is not like/not as bad as experiencing the pain	4	9.5
I would prefer not to forget parts of my life even if they are painful or bad	6	14.3

**Table 4:** Responses to second-tier Reason question for a preference for 1 hour of remembered pain, Experiment 2, where first-tier Reason was ‘10 hours of pain would be hard to bear’ (*n* = 15)

Reason	Frequency	%
I would be unable to bear the 10 hours of pain at the time when it is happening	4	26.7
It would be easier to endure the 1 hour of pain at the time when it is happening	5	33.3
Experiencing pain for 10 continuous hours might have long-term effects on my mind or body	5	33.3
I wouldn’t want to know afterwards that I suffered for 10 hours, even if I don’t remember experiencing the pain itself	1	6.7

**Table 5:** Responses to first-tier Reason question for a preference for 10 hours of never to be remembered pain, Experiment 2 (*n* = 29)

Reason	Frequency	%
It is less unpleasant to have had 10 hours of past pain I can’t remember than 1 hour that I can	12	41.4
There is nothing unpleasant for me about pain in my past that I will never remember, no matter how long it lasted, because it’s over and done with	7	24.1
There is nothing unpleasant for me about pain in my past that I will never remember, no matter how long it lasted, because if I can’t remember it then in some sense it didn’t happen to me	10	34.5

**Table 6:** Responses to second-tier Reason question for a preference for 10 hours of never to be remembered pain, Experiment 2, where first-tier Reason was ‘it is less unpleasant to have had 10 hours of past pain I can’t remember than 1 hour that I can.’ (*n* = 12)

Reason	Frequency	%
Lasting memories of pain would stay with me, and this is worse than undergoing more hours of pain that I can’t remember	8	66.7
Lasting memories of pain are more intense than undergoing the pain itself	0	0
If I remembered the pain, it might create long-term psychological effects	3	25.0
If I remembered the pain, it would make it difficult for me to cope with anticipating future pain in general	1	8.3

#### Discussion of Experiment 2

Experiment 2 produced substantial evidence that when asked what news they hope to hear about events in the past, there are notable individual differences in the relative weighting people assign to the disutility of pain as remembered (‘secondary’) and as experienced (‘primary’). First, the number of participants who preferred 1 hour of remembered pain did not differ significantly from those who instead preferred 10 hours of never to be remembered pain. Having had 1 hour of pain that will be remembered was the most frequently preferred scenario, but a substantial minority (around a third) hoped to hear that they had experienced 10 hours of pain that they would never remember. That there was no statistically significant difference between the proportion preferring 1 hour of remembered past pain and the proportion preferring 10 hours of never to be remembered pain replicates results from the equivalent condition of Experiment 1 (Past News), suggesting that in the previous experiment, apparent individual differences in preferences were not simply due to the absence of an ‘indifferent’ option. Second, a further substantial minority of participants was indifferent between 1 hour of remembered pain and 10 hours of never to be remembered pain. Third, individual differences were evident when participants were offered the possibility of switching their preference in the face of larger amounts of pain associated with their preferred treatment: substantial proportions of participants indicated that they would trade off at the smallest hypothetical increase; at the largest hypothetical increase; and never. We found no evidence that these individual differences were explained by participants’ self-reports of the severity of pain they had experienced in real-life. Finally, the reasons that participants gave for their preferences diverged substantially. Self-reported reasons can only be interpreted tentatively, particularly due to the small numbers selecting some options, but we consider some interpretations of the self-report findings in the General Discussion.

## General discussion

Future biases in hedonic preferences have attracted recent interest in experimental philosophy (see, e.g., Baron et al., 2023; Caruso et al., 2024; Greene et al., 2021, 2022a, b, 2024; Latham et al., 2021, 2023), and some findings have suggested that such preferences may be affected by the availability of memory for past experiences (Lee et al., 2022). In the light of that body of research, we suggested that one manifestation of a future bias may be that the experience of pain in the past has relatively little (dis)utility compared to memories that can continue to be experienced in the future. However, no study to date had directly examined this issue. Through two experiments, we probed the weight people assign to the disutility of secondary, recollective experiences of pain, in comparison to the disutility assigned to the experience of living through the painful events themselves. Our findings indicated that there are notable individual differences in the relative utility of memory for a painful past experience versus the primary experience of pain itself (Experiments 1 and 2). Just fewer than half of participants showed a preference for 1 hour of remembered past pain over 10 hours of never to be remembered pain (Experiment 1 [Past News condition] and Experiment 2), and even among those participants, there was substantial variability in when and whether they would shift preferences if the amount of remembered pain was increased (Experiment 2). However, people were more uniform in their judgments when the painful experience (and memories for it) were located in their future: they typically preferred to undergo less future primary pain regardless of whether it will subsequently be remembered, suggesting that they set aside or discount future memories of pain (Experiment 1).

### Future bias: responses to choices about the past

As described in our introduction, it has sometimes been suggested that people have an absolute future bias, where this has been specifically described as a tendency to completely discount past experiences (Sullivan, 2018). We can be relatively confident that the participants who had an initial preference for 1 hour of remembered pain over 10 hours of never to be remembered pain (around 40% in both Experiments) are not absolutely future biased, since they preferred the state of affairs that included future disutility in the form of memories of past pain. The trade-off task in Experiment 2 further showed that, whilst there was considerable variation across this subset of participants in the extent to which this preference persisted when the quantity of remembered past pain increased, roughly one-quarter of participants with the initial preference of 1 hour of remembered pain stated that they would only change their preference once both options involved the same magnitude of past pain (10 hours).

In this scenario, we can pull apart considerations regarding the duration of pain undergone and memory (or not) for such pain. For the participants in question, the quantity of pain associated with the primary experience itself appeared to be the overriding consideration. Any disutility associated with memories of pain merely served to tip the scales in the case where the amount of past pain did not differ between the options. Correspondingly, for those who initially declared a preference for 1 hour of remembered pain, but then traded off before the magnitudes of the primary experiences became equal, we can infer from their trade-off points something about the relative weightings they assigned to the disutility of primary and secondary experiences, respectively. We will return below to the more perplexing case of the further quarter of this subset of participants who stated they would never trade off remembered for never to be remembered pain, regardless of the quantity of past remembered pain.

Reflecting on responses to the forced choice questions, it is notable that some participants responded in ways that we would not expect from heavily future biased people. At the first-tier level, the most popular reason selected as best explaining a preference for 1 hour of remembered past pain was: ‘10 hours of pain would be hard to bear’ (see Table 3, and see Table 4 for the explanations favored by those participants who selected this as the reason for their preference). The second most popular reason selected was ‘It is less unpleasant to have had fewer total hours of pain’, followed by ‘A smaller number of hours of pain that I will remember would cause less unpleasantness for me than a larger number that I won’t ever remember’ as the third most popular selection. Each of these three responses provides evidence that participants are not simply considering future (dis)utility – i.e., that the participants making such selections do not make them on the basis of future bias. In fact, rather than only considering what would be best *for their future* from the standpoint of the present, these participants appear to be considering what would be best *for their lives*, where this takes into account past, present and future (dis)utility. These participants are plausibly adopting an impersonal perspective – what Scheffler (2021) calls ‘the whole-life perspective’, in contrast to ‘the future-facing perspective’ – and weighing the (dis)utility afforded by primary and secondary experiences regardless of their temporal location.

One additional complication is worth noting here. Of those who selected, at the first-tier level, ‘10 hours of pain would be hard to bear’ as the best reason for their preference for one hour of pain to be remembered, one-third (five participants) selected as the best explanation of this reason that ‘Experiencing pain for 10 continuous hours might have long-term effects on my mind or body’ (Table 4). Hence, despite the vignette specifying that neither form of Denbora Syndrome carried greater long-term health-consequences, and that neither form of radiation therapy carried a higher risk of long-term damage to one’s health, some participants nevertheless harbored concerns about future effects of having undergone a longer painful treatment, even granting that the treatment will not be remembered. Thus, there may still be a form of future bias – one that concerns such anticipated future consequences – at work in the responses of this group of participants.

Although a preference for 1 hour of remembered pain was the most common response in Experiment 2, in both experiments, a substantial proportion of participants instead chose 10 hours of never to be remembered pain over 1 hour of remembered pain. Participants’ responses to the forced choice questions in Experiment 2 about the reasons for their initial preferences provide some evidence that they attached disutility to remembering past pain in their future. At the first-tier level (see Table 5), the most popular reason selected for preferring 10 hours of pain never to be remembered was: ‘It is less unpleasant to have had 10 hours of past pain I can’t remember than 1 hour that I can’. Of the participants who selected this first-tier response, at the second-tier level (Table 6), which asked for the best available reason for their first-tier selection, the most popular choice was: ‘Lasting memories of pain would stay with me, and this is worse than undergoing more hours of pain that I can’t remember’. Thus, participants who preferred 10 hours of pain never to be remembered say that they are taking into account the disutility of memories of pain and tell us that, as a result of this, they would prefer the greater magnitude of past pain to less past pain accompanied by future memories, a pattern of responses that can be interpreted as indicating a bias towards the future and a sensitivity to the (dis)utility afforded by secondary experiences.

The findings of Experiment 2 potentially shed further light on the magnitude of such a bias, insofar as people were encouraged to report just how much past never to be remembered pain they would prefer to have when the alternative was 1 hour of past remembered pain. Most of the sub-set of participants who initially expressed a preference for 10 hours of unremembered pain shifted their preference when the amount of never to be remembered past pain approximately doubled, suggesting that past pain did indeed have some have disutility for them. For the trade-off task, we would expect absolutely future biased participants to indicate that they would accept any quantity of past never to be remembered pain over an hour of past remembered pain. Around one-quarter of participants who initially preferred 10 hours of never to be remembered pain did indeed claim that they would never trade off their preference; even a tenfold increase in never to be remembered pain (to 100 hours) did not induce them to do so. It is these participants that might most confidently be thought to discount past painful experiences absolutely. It should be noted, though, that they constituted only around 8% of the full sample in Experiment 2. Thus, while the overall pattern of results of both experiments are at least compatible with the idea that past painful experiences are discounted to some degree, at least by a notable proportion of participants, there is very little evidence that absolute discounting of past pain is widespread.

Experiment 2 allowed participants to express indifference between 1 hour of remembered pain and 10 hours of never to be remembered pain; around 20% of participants chose this option. For these participants, it is possible that 10 hours of never to be remembered past pain was taken to be more or less equivalent, in terms of the (dis)utility it affords, to 1 hour of past pain accompanied by subsequent memory for the event. If this was the case, we would expect the participants to trade off as soon as the ratio changed from 1:10 of remembered past pain to never to be remembered past pain. A minority of initially indifferent participants did adopt a preference at the minimum possible new duration of pain in the trade-off task (16.7% at 2 hours of past pain to be remembered; 38.9% at 20 hours of past pain never to be remembered). It would thus appear, for these participants, that it was something about the 1:10 ratio that initially led to their indifference between the two options. For those initially indifferent participants who traded off at some point (that is, did not indicate that they would never trade off), it may be that they initially found the comparative weightings of the two alternatives difficult because they found no clear temporal discounting rule to follow. However, a large minority of the initially indifferent participants stated that they would never have a preference regardless of the ratio of remembered to never to be remembered pain, and these participants may simply have seen the choice options as incommensurable. This serves as a reminder that we cannot assume that people will straightforwardly be able to, or want to, translate the types of choices presented to them in this type of task into quantities of utility.

Overall, our findings regarding choices about the past paint a picture of substantial individual differences, and give an indication of the reasoning and beliefs that inform them. Some people find choosing between two states of affairs, one involving a greater magnitude of past pain never to be remembered and one involving a lesser magnitude of past pain accompanied by memory for said pain, more difficult than others. Some people would rather encounter a lesser magnitude of remembered pain, and some would rather have a greater magnitude of pain if it is never to be remembered, indicating differences in the relative utility afforded to memories of pain relative to primary experiences of pain, and/or differences in the extent to which these participants appear to be biased towards the future. Even among those participants who had an initial preference for one of these two scenarios, there appear to be substantial differences in the weighting accorded to the magnitude of past pain relative to memory for pain, as indicated by the divergent trade-off points.

### Future bias: past-future differences

There was a clearer pattern of performance when participants were asked to make judgments about remembered versus never to be remembered pain in the future: in the Future conditions, participants rarely chose a greater amount of never to be remembered future pain and their responses differed significantly from those in the Past condition (Experiment 1). This difference between the two conditions could itself be taken as suggestive of a past-future difference in the level of (dis)utility associated with primary experiences. That is, one interpretation of the effect of tense in Experiment 1 is that the vast majority of participants assigned high (dis)utility to future experiences, whereas this was not uniformly the case when experiences were in the past. As noted in the Discussion of Experiment 1, though, such an interpretation of participants’ choices regarding the future is not completely straightforward. Just as we have suggested that the availability of memory may have an impact on the overall (dis)utility associated with past experiences, the availability of the secondary experience of anticipation may also have an impact on the overall utility associated with future experiences. In particular, in this study we focused on a painful future experience, and it is well-established that people are averse to the dread experienced when contemplating future unpleasant events (Berns et al., 2006; Hardisty & Weber, 2020; Loewenstein, 1987; Sun et al., 2022). Thus, it is possible that people’s preference for a smaller amount of remembered pain in the future reflects at least in part a belief that aversive dread would be experienced during the waiting period before treatment– and that the dread would be less for a smaller magnitude than a greater magnitude of anticipated pain– rather than reflecting simply the disutility attached to the primary experience of pain itself.

Our results in this preliminary study do not allow us to disentangle disutility associated with primary versus secondary future experiences, although the role of anticipatory secondary experiences is a theme emerging in current behavioral decision-making research on temporal discounting of future negative and positive outcomes (Molouki et al., 2019; Patt et al., 2024; Sun et al., 2022). Regardless of the source of the disutility for future never to be remembered pain, the effect of tense on our findings can potentially be seen as indicative of a type of short-termism in future-oriented decision making, insofar as it suggests that participants were not reaching their decision by thinking ahead to the point in the future in which the treatment would be over (i.e., be in the past), but by thinking about the immediate future in which the treatment would occur. Thus, one interpretation of the effect of tense is that it indicates that people may be prone to neglect or discount future memories of pain. One interesting question for future research may be to examine whether quite different patterns are obtained when the experience in question is positive rather than negative.

### The (dis)utility of memory

In addition to shedding light on people’s time biases, our results provide a first attempt at probing the degree to which different people understand secondary experiences– memories, in this context– as a source of (dis)utility in their own right, in addition to the (dis)utility of the events lived through. In Experiment 2, for those participants who initially preferred a remembered painful treatment lasting 1 hour, the trade-off task may provide some further insights into any disutility accorded to memories of pain. As we have already pointed out, for approximately one-quarter of these participants, their preference for remembered pain was maintained only until it reached 10 hours– i.e., a duration equal to that of the alternative state of affairs of 10 hours of never to be remembered pain. Such people might be said to accord little independent disutility to secondary experiences, such as the memory of pain, given their consistent preference for a lesser magnitude of pain undergone first-hand regardless of such memories.

A further one-quarter of these participants maintained that they would *never* trade off their initial preference for 1 hour of remembered pain. Far from according little independent disutility to memory, these latter participants’ responses suggest that for them, memory has some kind of instrumental or even intrinsic value, so much so that they would rather have memory for events experienced first-hand than not, even for memories of pain. There are at least two types of reasons why one might value memories in this way. First, there are potentially negative social associations with memory loss for particular periods of time, such as a distrust of those in control of such memory loss, worries about what may have occurred during the time in question, and a concern for being able to explain to others what occurred during notable life events. This would tie in with recent accounts of episodic memory that stress its role(s) in human social engagements (Mahr & Csibra, 2018). Second, even negative memories might be thought to have potentially positive sides to them, such as giving one a sense of improvement in one’s life, or opportunities to learn from past experience and to bond with others who have undergone the same or similar experiences (see Schechtman, 2022, for relevant discussion).

In the current experiments we tried to guard against such considerations by having the protagonist awake and alert during the procedure, only having memories erased after the event, and by having the pain be a consequence of a medical procedure to treat a newly discovered condition, rather than some arduous hardship that is to be overcome. However, this likely did not lead to *all* participants bracketing such concerns. In fact, in Experiment 2, of those participants who declared a preference for 1 hour of remembered pain over 10 hours of pain never to be remembered, approximately 15% selected as the best reason for their preference that they would prefer not to forget parts of their life, even if they are painful or bad (see Table 3). This raises the further possibility that, for some people, there are no ‘purely’ hedonic experiences (i.e., experiences whose value is given entirely by the amount of pleasure or displeasure yielded by the primary experience itself as and when it occurs), and that for these people, issues concerning the intrinsic and instrumental value of such secondary experiences will always complicate declared preferences in experiments such as ours. Future research could probe the nature and prevalence of some of the different ways in which people think of memory as playing into overall goods in their lives, such as quantity of (dis)utility, considerations about *types* of memory or *qualities* of memory, the role of judgements about the best overall ‘shape’ of a life, and concern with being able to recall events that they have undergone for the sake of recall itself.

However, participants’ answers to the forced choice questions suggest that not everyone attached value to memory per se. Of those participants with an initial preference for 1 hour of remembered pain, at the first-tier level (see Table 3), the reason ‘I would prefer not to forget parts of my life even if they are painful or bad’ was, perhaps surprisingly, only the fourth most popular reason. Moreover, while we have taken our study to be examining the relative disutility of primary experience versus memory, it is important to bear in mind that the de facto effect of any given memory on one’s wellbeing is likely to be closely linked to how often it is re-visited and the strength of the associated emotions it stirs up. Our preliminary findings that used a single scenario do not allow us to examine the extent to which participants considered such factors.

Asking for participants’ reasons for their preferences also yielded some information about the role that memories (of pain) are taken to play in something like one’s life history or personal identity over time. The reasons which could be selected as the best reason for preferring 10 hours of pain never to be remembered distinguished between discounting the past (‘because it’s over and done with’) and discounting what cannot be recalled (‘because if I can’t remember it then in some sense it didn’t happen to me’). A substantial proportion of participants who saw this question selected the latter reason. This may provide some defeasible support for the claim that (at least some) people naively operate with a broadly psychological criterion of personal identity over time, insofar as they do not consider events they cannot remember to be part of their identity, and this may feed into their biases. (For recent work on issues concerning identity and the self in experimental philosophy, see the contributions in Tobia, 2022.) Conceptions of the self, and the self’s relation to past and future selves (or ‘time-slices’), as well as valuations of goods for such selves, will plausibly influence people’s time-biases, as will people’s first-order ethical views; this is not something which we were able to probe in the current context, but would be an interesting and potentially fruitful avenue for future empirical work.

## Concluding remarks and future directions

Our study presents a first attempt at directly addressing the relative (dis)utility people assign to a past primary experience versus memory for that experience, while also probing the extent to which such weightings modulate people’s temporally asymmetric hedonic preferences. Overall, through two experiments, our findings paint a picture of substantial individual differences, both concerning people’s discounting of past pains and concerning the relative weightings people assign to the (dis)utility of primary and secondary experiences of pain. Thus, any generalizations about a (level of) future bias shared across a population – or concerning the degree to which people weigh the (dis)utility of primary experiences relative to recollective experiences – would appear to be premature.

Given the limited research on the (dis)utility of memory and how this contributes to and structures people’s past-future hedonic preferences, there are various further questions to be pursued both in empirical research in psychology and in philosophy. Whilst this paper has focused in particular on pain and memories of pain, one area ripe for further research concerns the relation between the phenomenology and utility of primary and secondary experiences quite generally. How might thinking about the phenomenology of experiential memory help us to conceptualize the (un)pleasantness of memories of pain and pleasure, and the (dis)utility that such memories afford? For example, are there marked differences between the relative degrees of (un)pleasantness in the primary and secondary experiences for pleasurable and painful events? And does this vary as a function of the way in which the (un)pleasantness of the secondary experience is inherited from the corresponding primary experiences? Future work might also aim to explicitly probe the different valuations people may make of memories of pain, from being a source of disutility to providing opportunities for learning and feeding into overall judgements regarding what makes for a better life.

Another respect in which the scope of this paper has been restricted is that the only type of secondary experience it has considered were memories. But, as we have pointed out, important questions also arise with respect to future-oriented secondary experiences and emotions such as dread, and how these interact with and structure people’s time biases. Two further lines of inquiry might be of particular interest in this context. First, it might be necessary to make a more fine-grained distinction between the secondary experience of anticipating a primary experience, and that of anticipating a waiting period (see, e.g., Sun et al., 2015), given that waiting is often a distinctive part of future-oriented choices. Second, and relatedly, when focusing upon experiences said to occur in the future and the secondary experiences they may afford, there may also be a role for consideration of what we might term tertiary experiences – not simply anticipating the first-hand experiences of pleasure/pain, but anticipating the memories that such experiences may afford, or anticipating the anticipatory emotions one might feel during a waiting period.

In summary, our findings provide some initial insights into the relative utility of primary experiences and secondary (specifically memory) experiences, and how this might contribute to past-future hedonic preferences. However, perhaps more importantly, they also point to an issue – the level, source, and nature of utility of secondary experiences – that appears to be ripe for further investigation and theorizing. Our empirical approach provides an initial way of addressing this issue.
